# Convolutional neural network-based classification of cervical intraepithelial neoplasias using colposcopic image segmentation for acetowhite epithelium

**DOI:** 10.1038/s41598-022-21692-5

**Published:** 2022-10-14

**Authors:** Jisoo Kim, Chul Min Park, Sung Yeob Kim, Angela Cho

**Affiliations:** 1 Center for Artificial Intelligence, Korea Institute of Science and Technology, 5 Hwarangro14-gil, Seongbuk-gu, Seoul, 02792 Republic of Korea; 2 Department of Obstetrics and Gynecology, Jeju National University Hospital, Aran 13gil 15 (Ara-1Dong), Jeju City, 63241 Jeju Self-Governing Province Republic of Korea

**Keywords:** Translational research, Cervical cancer

## Abstract

Colposcopy is a test performed to detect precancerous lesions of cervical cancer. Since cervical cancer progresses slowly, finding and treating precancerous lesions helps prevent cervical cancer. In particular, it is clinically important to detect high-grade squamous intraepithelial lesions (HSIL) that require surgical treatment among precancerous lesions of cervix. There have been several studies using convolutional neural network (CNN) for classifying colposcopic images. However, no studies have been reported on using the segmentation technique to detect HSIL. In present study, we aimed to examine whether the accuracy of a CNN model in detecting HSIL from colposcopic images can be improved when segmentation information for acetowhite epithelium is added. Without segmentation information, ResNet-18, 50, and 101 achieved classification accuracies of 70.2%, 66.2%, and 69.3%, respectively. The experts classified the same test set with accuracies of 74.6% and 73.0%. After adding segmentation information of acetowhite epithelium to the original images, the classification accuracies of ResNet-18, 50, and 101 improved to 74.8%, 76.3%, and 74.8%, respectively. We demonstrated that the HSIL detection accuracy improved by adding segmentation information to the CNN model, and the improvement in accuracy was consistent across different ResNets.

## Introduction

Cervical cancer is the fourth most common cause of death in women worldwide^1^. Early screening for cervical cancer reduces morbidity and mortality because it is slow-growing and progresses through precancerous lesions^2^. The precancerous lesions of cervical cancer are cervical intraepithelial neoplasias (CINs), which can be divided into low grade squamous intraepithelial lesions (LSIL), such as CIN1, and high-grade squamous intraepithelial lesions (HSIL), such as CIN2 and CIN3^3^. Women with HSIL require surgical management, whereas those with LSIL need conservative observation^3,4^.

Currently, cervical cytology (i.e., Papanicolaou or Pap test) and the test for presence of human papillomavirus are used to screen for cervical cancer. If there is an abnormality in the screening test, colposcopy is performed to identify cervical lesions using low magnification microscopy with acetic acid and Lugol’s solution^5^. Several colposcopic findings, such as dense acetowhite epithelium, coarse mosaic, punctation, and atypical vessels, are considered abnormal^6^. Because these findings suggest HSIL or invasive cancer, guided colposcopic biopsy is required.

Colposcopy requires experienced colposcopists, and interobserver variability is considered the main limitation of this test. The sensitivity of colposcopy in detecting CINs varies from 81.4 to 95.7%, with a specificity of 34.2–69% even when performed by experienced colposcopists^7–10^. In addition, in low- and middle-income countries, the diagnostic accuracy of colposcopy is reported to be relatively low, ranging from 30 to 70%^8,11^. In these circumstances, the need for interpretation of colposcopy using artificial intelligence has emerged, to improve diagnostic accuracy.

Diagnosing CINs objectively based on clear rules using only image data is difficult; thus far, the need for biopsy and its location has been determined based on subjective opinion of experts. Because this approach depends on the abilities of experts, improving accuracy through existing artificial intelligence methods, such as rule-based method, is difficult. However, with the recent developments in deep learning technology, attempts have been made to apply it to detect CINs using subjective findings.

Deep learning is defined as machine learning algorithms that attempt high-level abstraction through a combination of several nonlinear transformation methods^12^. Artificial neural networks (ANNs) are used to implement deep learning, and the most representative example is a convolutional neural network (CNN). CNN, which consists of several hidden layers, such as convolutional, pooling, and fully connected layers, was first proposed by LeCun et al.^13^. With the lots of hidden layers, a CNN can extract the features of data, similarly to the human eye.

CNNs have been used in various applications to analyze images^14^, sounds^15^, and waves because they have achieved better performance than conventional data classification methods. In addition, currently a CNN is used for segmentation tasks, i.e., performs sematic classification for each pixel. This segmentation technique can be applied to find specific lesions in medical images.

Several studies have used CNNs for classifying colposcopic or cervicographic images. They primarily investigated the use of clinical information, such as age or Pap smear results, for classification^7,11,16,17^. In this study, we attempted to classify colposcopic images using only the image without any additional clinical information. Furthermore, no studies have been reported on using the segmentation method to classify CINs. We attempted to extract the segmentation results for acetowhite lesion as an image and use it for classification. The purpose of this study was to examine whether the accuracy of a CNN model in detecting HSIL from colposcopic images can be improved when segmentation information for acetowhite epithelium is added.

## Methods

### Data resource and data labeling

The Institutional Review Board (IRB) of Jeju National University Hospital approved this research (IRB number: 2022-02-004). The study was performed in accordance with relevant guidelines and regulations in compliance with the Declaration of Helsinki. The IRB waived the requirement of informed consent due to the retrospective nature of study, which was based on medical records. All colposcopic images taken by board-certified gynecologic oncologists in Jeju National University Hospital from 2008 to 2021 were retrospectively collected. All photographs were taken after applying acetic acid during colposcopy under white light. The images were captured using an Olympus OCS 500 colposcope with an Olympus CLV-S190 (Olympus Medical System Corporation, Tokyo, Japan). The size of all the images was 640 × 480 pixels.

The histopathologic results from colposcopy guided biopsy or loop electrosurgical excision procedure (LEEP) of all patients were reviewed. If the punch biopsy result and the surgical pathologic result following LEEP were different, the higher-grade lesion was selected. We aimed to differentiate images of lesions requiring treatment from those not requiring treatment. We labeled images of patients with normal to LSIL findings, which can be monitored without intervention, as “normal.” HSILs requiring surgical treatment such as LEEP were labeled as “abnormal.” The images of patients who did not undergo biopsy were labeled as “normal” if normal colposcopy findings were clearly recorded in their medical records. For patients who underwent biopsy, chronic cervicitis and CIN1 were labeled as “normal,” and CIN2 and CIN3 lesions were labeled as “abnormal.”

Adenocarcinoma in situ and invasive carcinoma were excluded due to their small numbers. Photos without biopsy were excluded if the colposcopy result was not specified as “normal” in the medical record. Images that did not include the cervix were also excluded from the dataset. A total of 3699 photographic images including 1385 normal colposcopic findings without biopsy, 579 chronic cervicitis, 722 CIN1s, 404 CIN2s, and 609 CIN3s proven by biopsy were used for training. A total of 2686 images were labeled as “normal,” and 1013 images were labeled as “abnormal.”

### Construction of datasets and data preprocessing

Because a method of combining two trained CNNs was applied, two datasets were constructed and used in this study. One dataset was used for training the CNN for classification, and the other dataset was used for training CNN for segmenting acetowhite epithelium.

**Figure 1:**
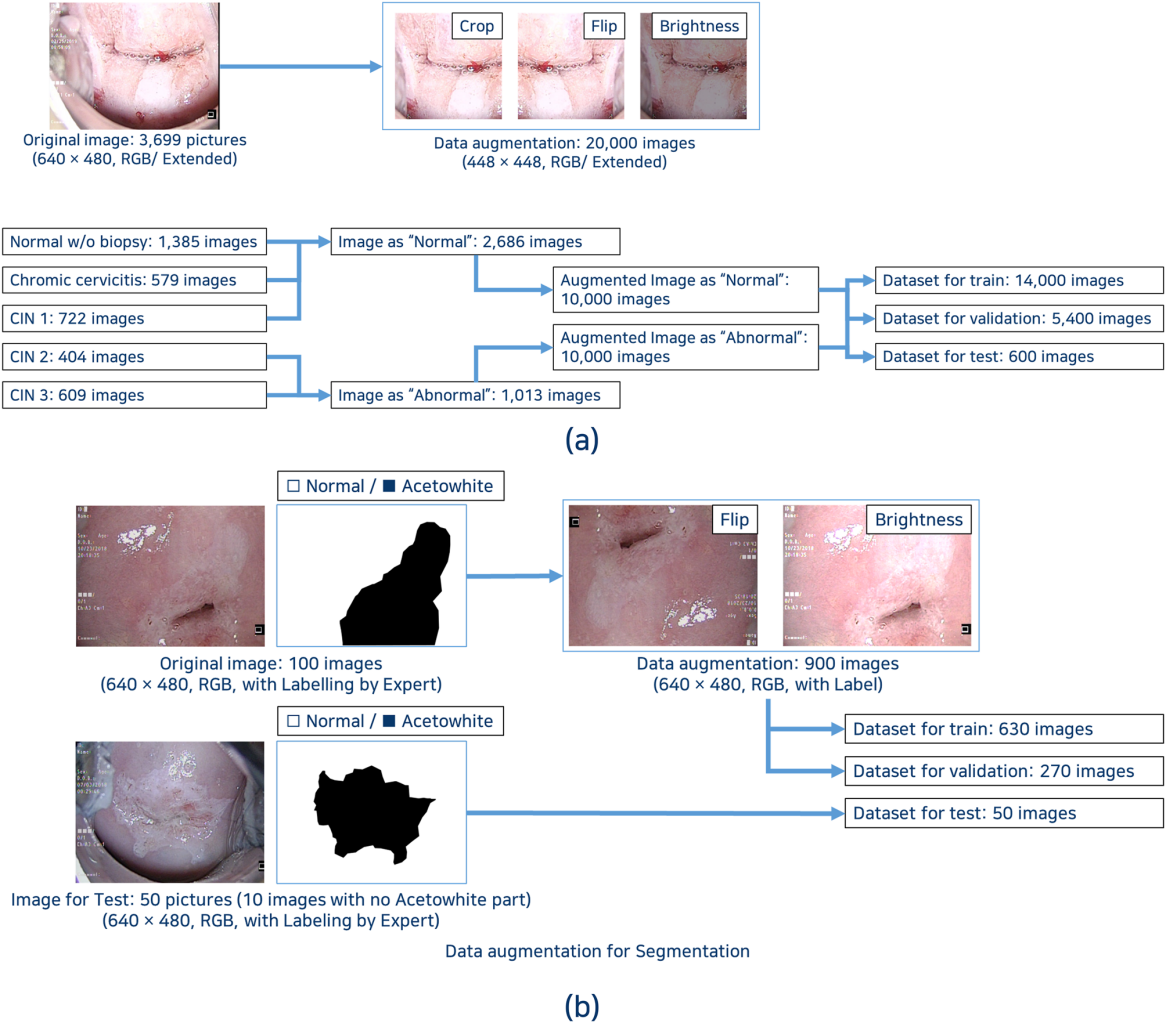
Construction of dataset (**a**) Dataset for image classification (**b**) Dataset for image segmentation.

The original data were augmented to increase the size and diversity of the classification dataset. The collected 3699 images were extended to 20,000 images by random cropping, flipping, and brightness tuning. From these augmented images, 14,000 images were used for training, 5400 images for validation, and 600 images for testing (Fig. 1a). In general, dataset for train/validation/test is divided at a ratio of 70/15 / 15, but in this study, the ratio was set to 70/27/3 in order to maximize the images required for training and validation with a limited number of images.

For segmentation, 100 images of the 3699 images that included dense or thin acetowhite epithelium and normal cervix without acetowhite lesions were labeled for acetowhite lesion by experts. Then, the images were augmented to obtain 900 images by random flipping and brightness tuning. From these augmented images, 630 images were used for training and 270 images were used for validation. For robustness, we used a test set constructed from the remaining images rather than from the 100 original images. Thus, another 50 images from the 3699 images were marked for acetowhite epithelium by experts and used as the test set (Fig. 1b).

### Classification and segmentation architecture

**Figure 2:**
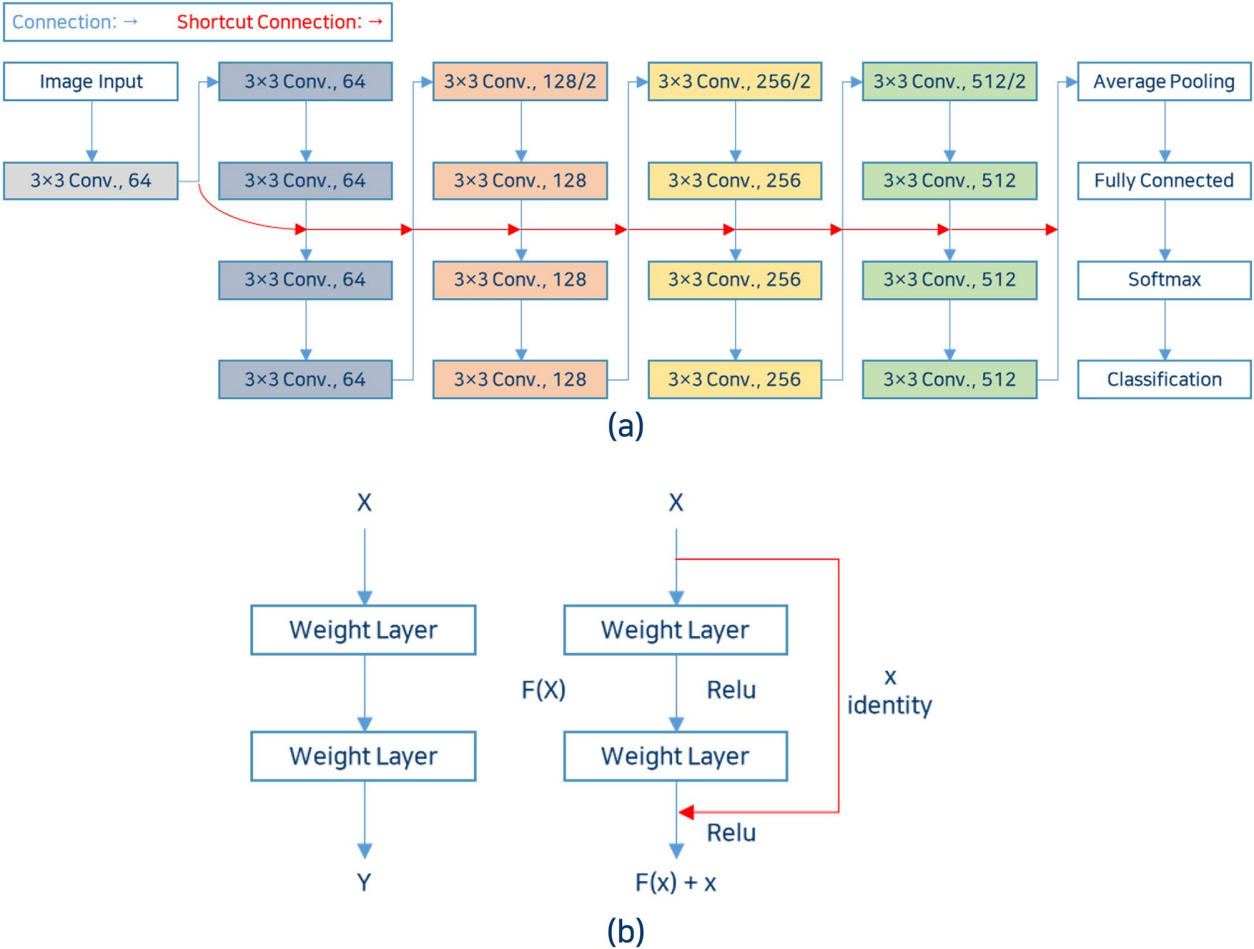
ResNet architecture (**a**) Neural network structure of ResNet-18 (**b**) Residual learning: building block.

We used ResNet for image classification (Fig. 2a). Figure 2b presents an existing CNN method used to find the optimal value of the input x through the learning layer, and ResNet used a method to find the optimal F(x) + x by adding the input x after the learning layer. Because this approach reduces both network complexity and gradient problems, it provides faster training^18^. The ResNets used in this study were ResNet-18, ResNet-50, and ResNet-101 according to the number of layers, and the higher the number, the deeper the neural network. With a deeper neural network, better classification performance is achieved on a larger dataset. However, due to the limited amount of data in this study and the binary classification task of feature regions in which target image diversity is not high, a deeper neural network may not necessarily guarantee better performance. Therefore, ResNets with various depths were used.

**Figure 3:**
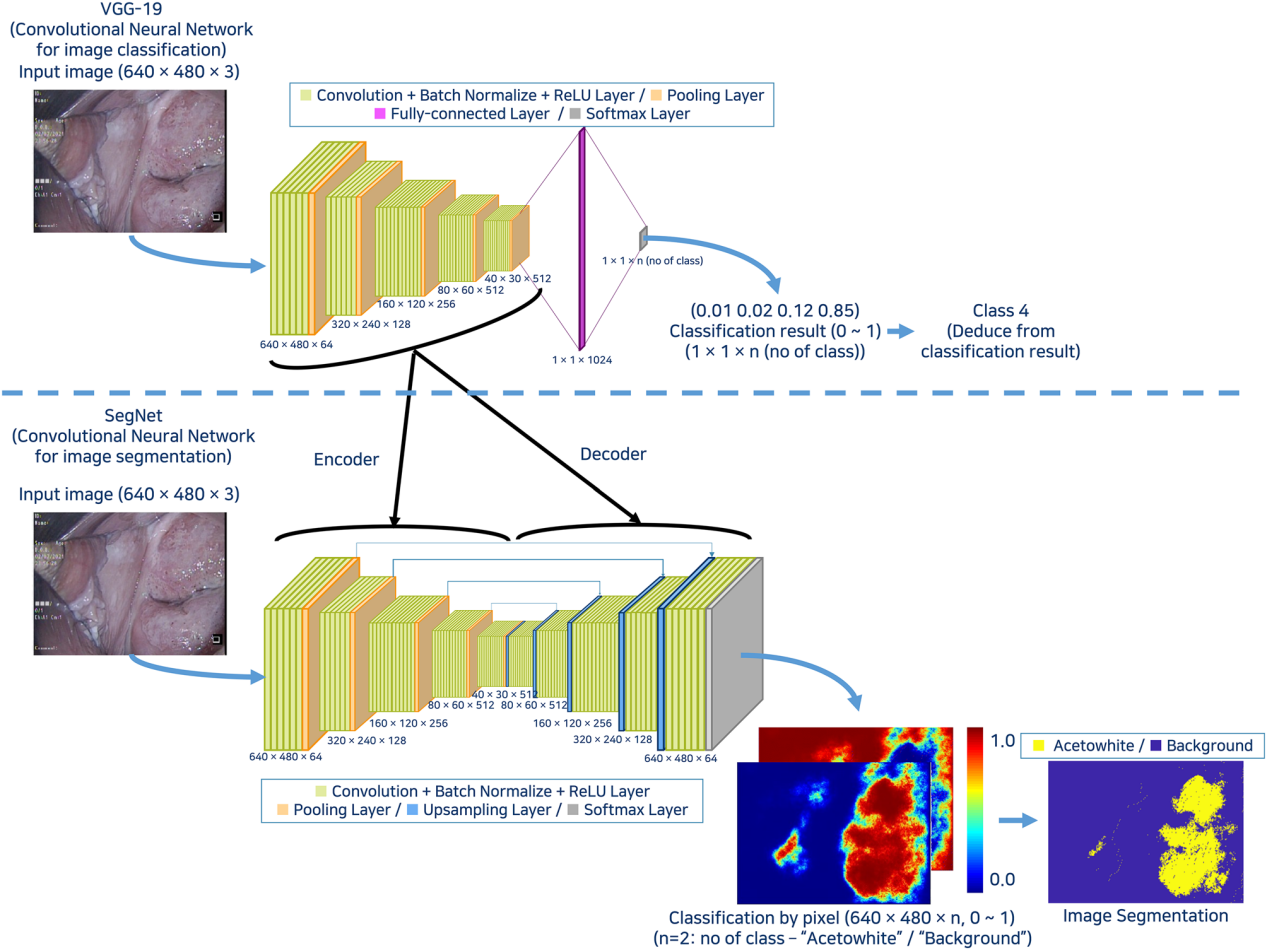
Architecture of VGG-19-based SegNet.

Acetowhite epithelium varies from thin to dense white lesions after applying acetic acid during colposcopy, and it is determined based on the subjective judgment of experts. Thus, it is difficult to automatically extract and digitize acetowhite lesions using rule-based methods. To address this, this study applied an image segmentation method using a trained CNN. A neural network trained on labeled images can automatically extract a specific part on its own, and thus the regions with acetowhite in the image data can be segmented and classified. SegNet is a common image segmentation method with an encoder-decoder structure that removes fully connected layer from the existing CNN (VGG-16 or VGG-19) and rearranges them in a symmetrical form. The role of the encoder network is to extract feature map of image, and role of the decoder network is to map the feature map extracted by the encoder to original resolution of input image for the pixel-wise classification^19^. In this study, a SegNet based on VGG-19 was used as the neural network for image segmentation to detect acetowhite lesions from image data (Fig. 3).

### Classification model concept

**Figure 4:**
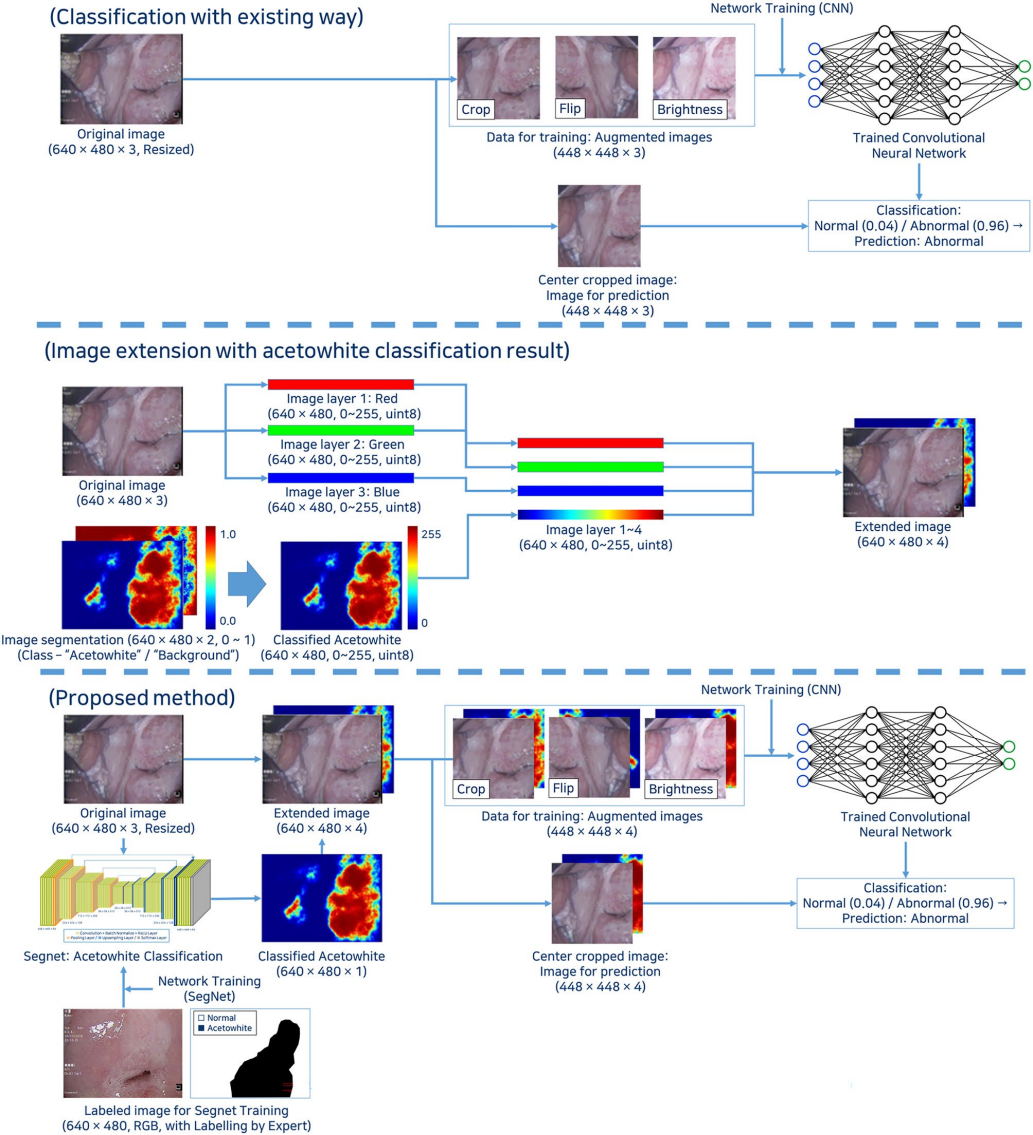
Concept diagram of the developed classification system.

Initially, we classified the images from the augmented dataset into normal and abnormal groups. Then, the images showing segmentation results of acetowhite lesions were merged with the original images and trained for classification again (Fig. 4). Acetowhite lesions were detected using the trained SegNet. The extended image data (4ch) were composed of the original image data (RGB, 3ch) and the detection about acetowhite lesions (1ch). The extended data were used for training the neural network and for classification. To augment the dataset, random translation, flipping, and brightness modification were applied. With the augmented dataset, the neural network was trained, validated, and tested.

### Development environment

Classification system was developed with Matlab 2021b with deep learning toolbox and parallel computing toolbox. Computer used as training facilitated with Intel^®^ Xeon^®^ Gold 6148 as CPU, 2 set of Tesla V100-SXM2 (RAM: 32 GB) as GPU. RAM of system is 128 GB. Operating system was Windows Server 2022 Standard.

### Definition of terms

**Figure 5:**
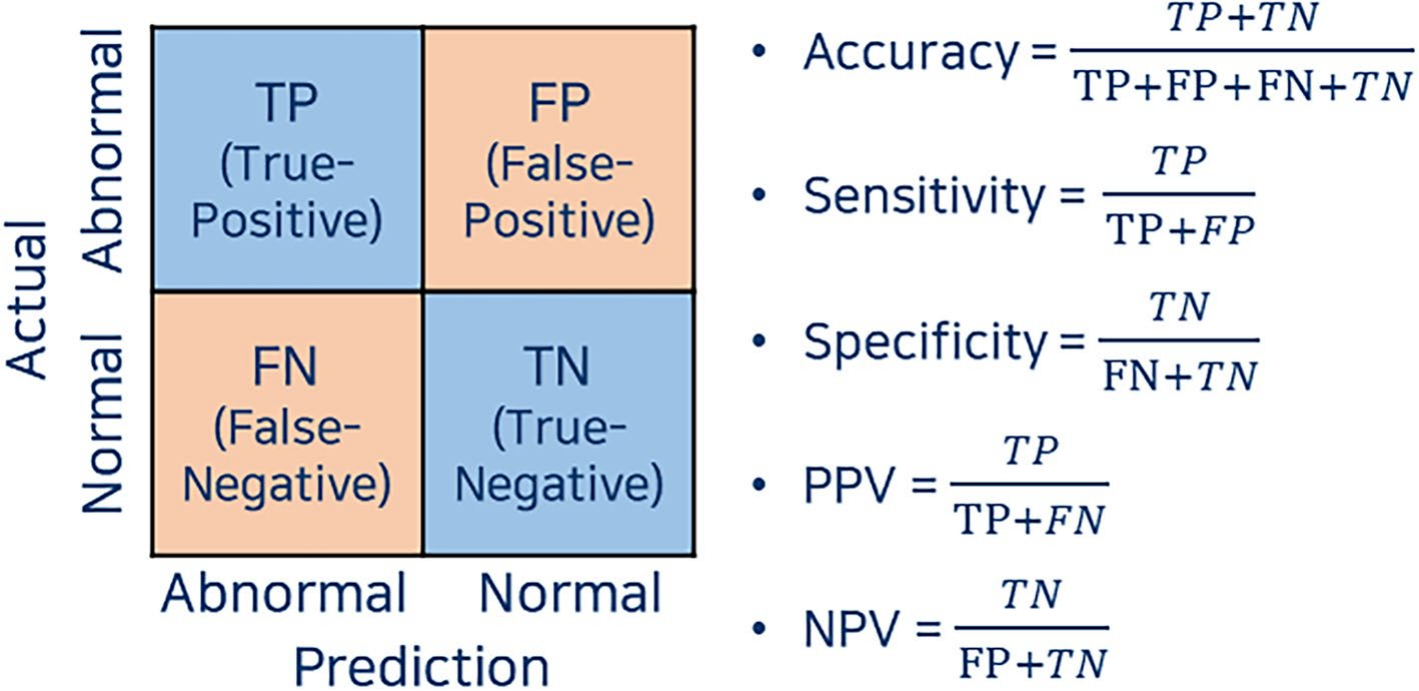
Definition of terms.

Accuracy is defined as the proportion of correct classifications among all classifications. Sensitivity, specificity, positive predictive value (PPV), and negative predictive value (NPV) are defined in Fig. 5. To provide a reference value, two board-certified gynecologic oncologists blindly classified the images from the same test sets as used for neural network evaluation.

### Gradient-weighted class activation map (Grad-CAM)

As applications related to classification using artificial intelligence and CNNs have been developed, researchers and users are experiencing difficulties in understanding how the algorithms operate, which is also known as the “black box” problem. This represents a considerable obstacle in research related to artificial intelligence and CNNs, and the need for eXplainable Artificial Intelligence (XAI) has progressed. Grad-CAM is the most common method to address this problem^20^. Grad-CAM uses the gradient of the target concept to create an approximate localization map that flows into the final convolutional layer and highlights areas of the image that are important for predicting the concept^21^. Through Grad-CAM analysis, it is possible to understand how a trained CNN works by identifying which parts of an image are responding as a feature to which label.

## Results

### Classification without segmentation information

ResNet-18, 50, and 101 achieved classification accuracies of 70.2%, 66.2%, and 69.3%, respectively. The experts classified the same test set with accuracies of 74.6% and 73.0%. The CNNs achieved a sensitivity of 61.8–65.8% for HSIL detection, and the experts achieved 67.8% and 71.4%.

### Segmentation of acetowhite lesion using SegNet

**Figure 6:**
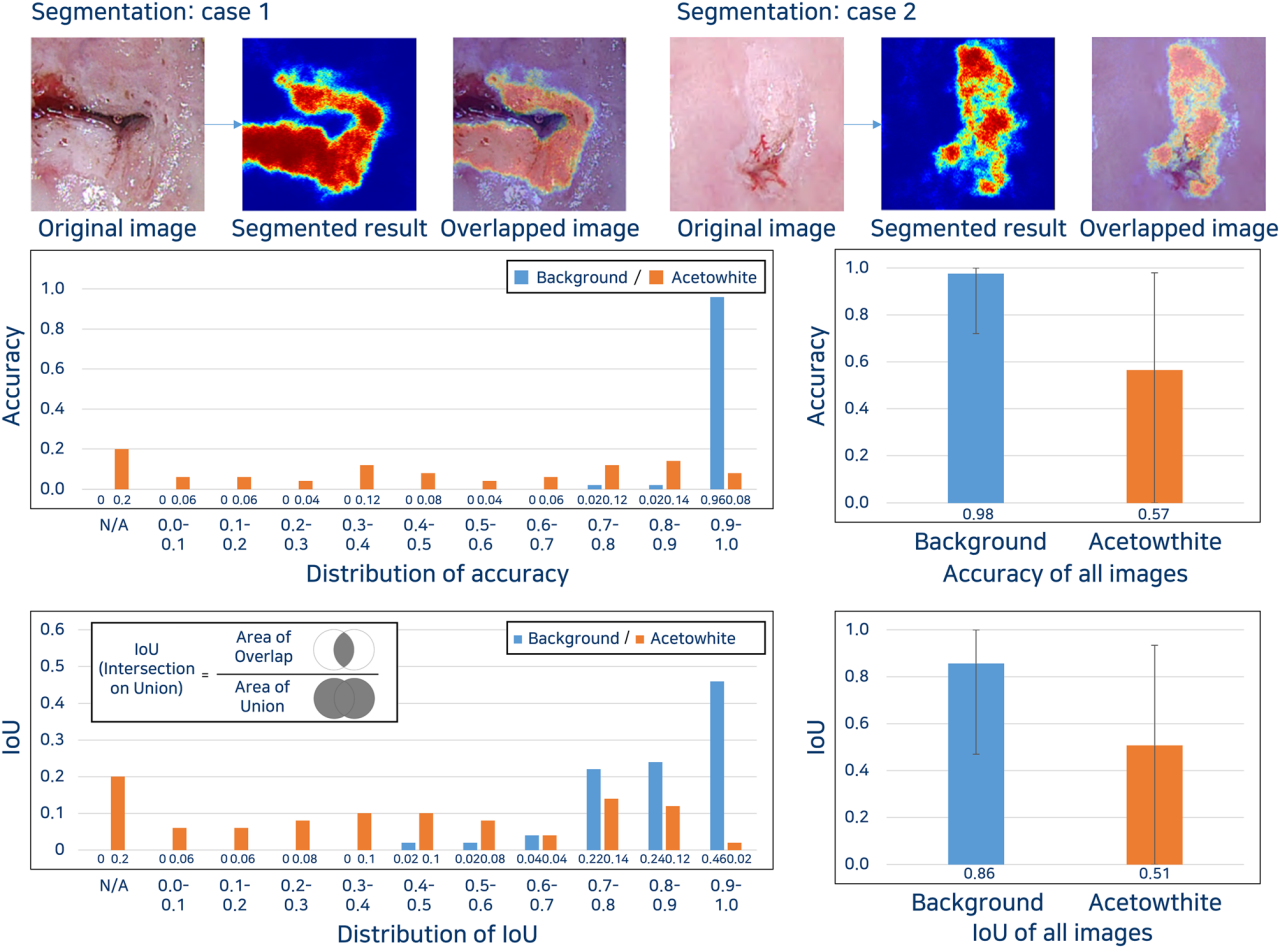
Segmentation performance of SegNet for acetowhite lesion.

Figure 6 shows examples of segmentation results consist of distribution of accuracy and the intersection over union (IoU). The average of accuracy of acetowhite epithelium and background were 0.57 and 0.98, respectively. The average IoUs of acetowhite epithelium and background were 0.51 and 0.86, respectively. Cases for comparison between segmentation results and marking for acetowhite lesion by expert presents in Supplementary Fig. S1.

### Classification with segmentation information

**Figure 7:**
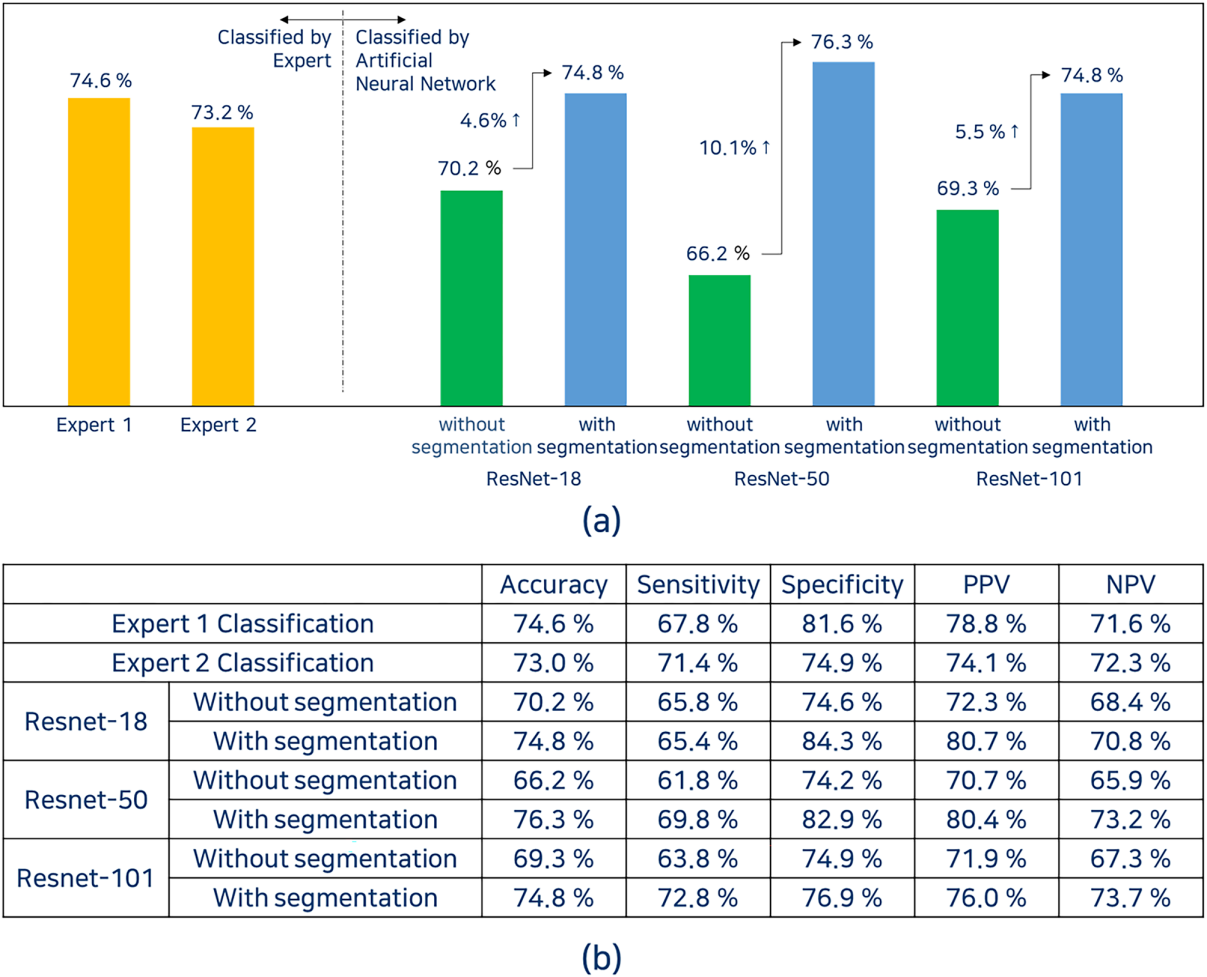
Classification performance (**a**) Classification performance with or without segmentation (**b**) Detailed evaluation results of the classification performance.

After adding segmentation information of acetowhite epithelium to the original images, the classification accuracies of ResNet-18, 50, and 101 improved to 74.8%, 76.3%, and 74.8%, respectively (Fig. 7a), with ResNet-50 achieving the most improvement (by 10.1%). For HSIL detection, ResNet-18, 50, and 101 achieved sensitivities of 65.4%, 69.8%, and 72.8%, respectively. It tended to be higher for deeper neural networks. ResNet-18, 50, and 101 achieved specificities of 84.3%, 82.9%, and 76.9%, respectively (Fig. 7b).

### Grad-CAM

**Figure 8:**
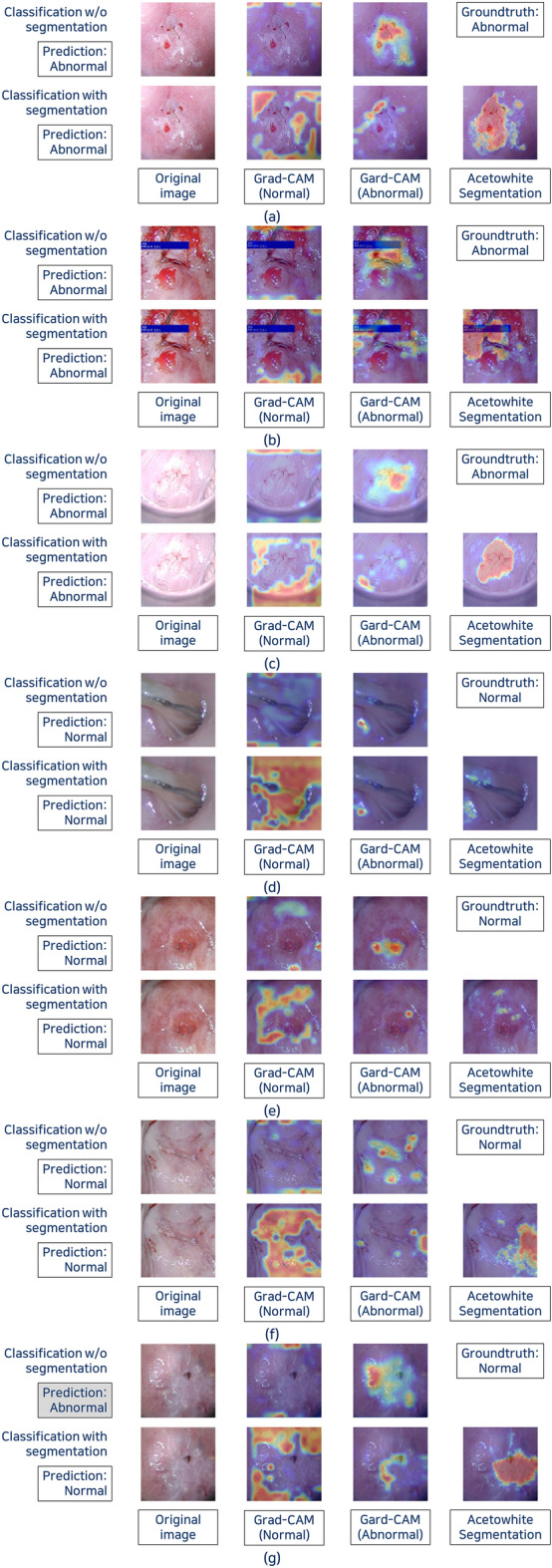
Examples of gradient-weighted class activation map (Grad-CAM) of classification. (**a**) to (**c**): Abnormal cases / (**d**) to (**g**): Normal cases.

Because the classification in this study is binary (normal and abnormal), it may have a different pattern from general multi-class classification. Therefore, reviewing Grad-CAM for all classes is expected to create an in-depth understanding of the operation of the corresponding neural network. Figure 8 presents examples of Grad-CAM showing the parts of the image in which the neural network finds normal and abnormal features.

## Discussion

We investigated whether HSIL of the cervix can be detected using deep learning-based algorithms and whether their accuracy can be improved by adding acetowhite lesion segmentation. Deep learning-based HSIL detection has achieved an accuracy between 63 and 84%^7,16^. In the present study, the accuracy of the classification using CNNs without segmentation information was 66.2–70.2%, which is relatively low. We speculate that this is because, unlike previous studies, we used not only unfocused colposcopic images but also poor-quality photos to reflect real clinical situations. In particular, when compared with a study using cervicography images, our colposcopic images may be of lower quality because cervicography requires to take picture according to set rules such as the cervix must be centered, and the proportions must be constant^22^. Using only post-acetic acid images may have been the cause of lower accuracy compared to studies using both pre- and post-acetic acid images^3,23^. Moreover, we used only images for training without any other clinical information, such as age, cervical cytology, and human papillomavirus, whereas the classification model proposed by Yuan et al. was trained using such information^7^.

A review of Grad-CAM could be helpful in explaining how an CNN works. Few studies have reported Grad-CAM results, but not in detail^16,17^. Most of the Grad-CAMs in previous studies were focused on the center of the cervix, which is largely consistent with our Grad-CAMs. However, according to our Grad-CAM in classification without segmentation, the deep learning models tended to focus on the area that included acetowhite epithelium to detect HSIL (Fig. 8a,b,c). This novel finding indicates that the models classified images in a similar way to colposcopists, and Grad-CAM could be used to find accurate biopsy sites in low-income countries, where experts are scarce.

By comparing the Grad-CAM before and after adding segmentation information, it is possible to hypothesize how the CNN classified the images. When segmentation information was not provided, the CNN detected by weighting the acetowhite lesion or the center of the cervix as abnormal features. However, when segmentation of acetowhite lesion was provided, the CNN already judged it as abnormal based on the segmentation information and did not appear to actively search for abnormal features (Fig. 8). Instead, it seemed to assign weights to the remaining parts as normal features. We posit that this might be how CNNs make time-efficient classifications. In addition, even with images that included a blue bar, the CNN seemed to ignore the noise (Fig. 8b).

We demonstrated that the HSIL detection accuracy improved by adding segmentation information to the CNN model, and the improvement in accuracy was consistent across different ResNets. Although a previous study attempted to segment acetowhite epithelium^7^, our research is the first to classify CINs using CNNs by merging images from segmentation results. The accuracy of our segmentation results was comparable with previous researches for segmentation of which IoU values of around 0.6 were considered relatively accurate^19,24^. Although these previous studies did not target segmentation for colposcopic images, our segmentation module has enough performance to deduce the tendency of acetowhite lesion. It can be applied to real clinical practice if the accuracy is improved through future studies. In addition, further studies to segment mosaicism or punctuation on colposcopic images using artificial intelligence should be conducted to provide more information.

The present study has a few limitations. Although we have provided expert classifications as reference values, we could not determine the superiority of the artificial intelligence classification because this study was not designed to directly compare them. In addition, the colposcopic images we used for training were photos with a speculum or cotton swab in them, taken in real clinical situations. Furthermore, part of the cervix may have been cut off during cropping as the cervix was not always centered in the photos, thus suspected HSIL lesions might not be included within the range of cropped picture. Although this may reduce accuracy of reading, this was inevitable because cropping is a data augmentation method that is essential for most CNN training processes.

However, our study has several strengths. Firstly, the above mentioned aspects of the images might have better reflected real-world conditions, which will be useful when this CNN model is actually applied in the future. Moreover, unlike previous studies, our classification model could be explainable through a review of the Grad-CAM. Finally, this study is the first to add segmentation information to classification.

## Conclusion

We developed a CNN-based classification model for HSIL detection using image segmentation for acetowhite epithelium, which mimicked physician’s decision-making during colposcopy. Using only images captured during colposcopy, we attempted to classify CINs without manually inputting other clinical information. Adding image segmentation of acetowhite lesions improved the accuracy of HSIL detection, achieving results comparable to colposcopy experts.

## Supplementary Information

Supplementary Information 1.

## Data Availability

The datasets analysed during the current study are not publicly available due to protection of personal information but are available from the corresponding author on reasonable request.
