# Supplementary figures and images for: Convolutional neural network-based classification of cervical intraepithelial neoplasias using colposcopic image segmentation for acetowhite epithelium
Source: Sci Rep. 2022 Oct 14;12:17228. doi: 10.1038/s41598-022-21692-5 (PMC9568549; doi:10.1038/s41598-022-21692-5)

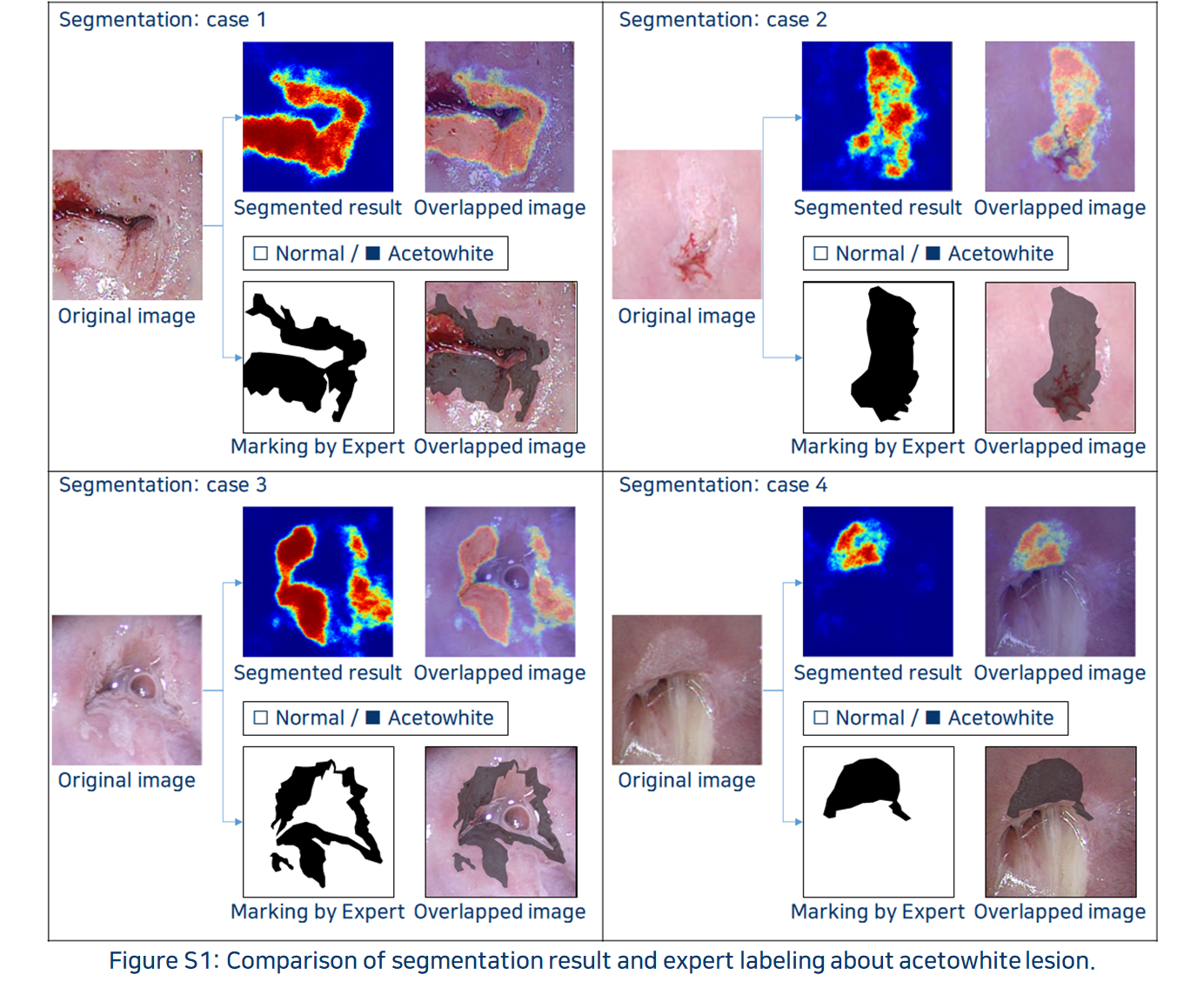

Supplement: Supplementary file 1 — Supplementary Information 1. [file 41598_2022_21692_MOESM1_ESM.png]
